# Surveillance of artemether-lumefantrine associated *Plasmodium falciparum* multidrug resistance protein-1 gene polymorphisms in Tanzania

**DOI:** 10.1186/1475-2875-13-264

**Published:** 2014-07-09

**Authors:** Reginald A Kavishe, Petro Paulo, Robert D Kaaya, Akili Kalinga, Marco van Zwetselaar, Jaffu Chilongola, Cally Roper, Michael Alifrangis

**Affiliations:** 1 Kilimanjaro Christian Medical University College and Kilimanjaro Clinical Research Institute, Moshi, Tanzania; 2 National Institute for Medical Research, Tukuyu Centre, Mbeya, Tanzania; 3 London School of Hygiene and Tropical Medicine, London, UK; 4 Department of International Health, Centre for Medical Parasitology, Immunology & Microbiology, Faculty of Health and Medical Sciences, University of Copenhagen, Copenhagen, Denmark

**Keywords:** *Plasmodium falciparum*, pfmdr1, Anti-malarial drug resistance, Artemether-lumefantrine, Tanzania, Polymorphisms, Malaria, Molecular markers

## Abstract

**Background:**

Resistance to anti-malarials is a major public health problem worldwide. After deployment of artemisinin-based combination therapy (ACT) there have been reports of reduced sensitivity to ACT by malaria parasites in South-East Asia. In Tanzania, artemether-lumefantrine (ALu) is the recommended first-line drug in treatment of uncomplicated malaria. This study surveyed the distribution of the *Plasmodium falciparum* multidrug resistance protein-1 single nucleotide polymorphisms (SNPs) associated with increased parasite tolerance to ALu, in Tanzania.

**Methods:**

A total of 687 *Plasmodium falciparum* positive dried blood spots on filter paper and rapid diagnostic test strips collected by finger pricks from patients attending health facilities in six regions of Tanzania mainland between June 2010 and August 2011 were used. Polymerase chain reaction-restriction fragment length polymorphism (PCR-RFLP) technique was used to detect *Pfmdr1* SNPs N86Y, Y184F and D1246Y.

**Results:**

There were variations in the distribution of *Pfmdr1* polymorphisms among regions. Tanga region had exceptionally high prevalence of mutant alleles, while Mbeya had the highest prevalence of wild type alleles. The haplotype YFY was exclusively most prevalent in Tanga (29.6%) whereas the NYD haplotype was the most prevalent in all other regions. Excluding Tanga and Mbeya, four, most common *Pfmdr1* haplotypes did not vary between the remaining four regions (*χ*^2^ = 2.3, p = 0.512). The NFD haplotype was the second most prevalent haplotype in all regions, ranging from 17% - 26%.

**Conclusion:**

This is the first country-wide survey on *Pfmdr1* mutations associated with ACT resistance. Distribution of individual *Pfmdr1* mutations at codons 86, 184 and 1246 varies throughout Tanzanian regions. There is a general homogeneity in distribution of common *Pfmdr1* haplotypes reflecting strict implementation of ALu policy in Tanzania with overall prevalence of NFD haplotype ranging from 17 to 26% among other haplotypes. With continuation of ALu as first-line drug this haplotype is expected to keep rising, thus there is need for continued pharmacovigilance studies to monitor any delayed parasite clearance by the drug.

## Background

*Plasmodium falciparum* multidrug resistance protein-1 (*Pfmdr1*) is an adenosine triphosphate-binding cassette protein located on the parasite’s food vacuole [1]. Mutations in the *Pfmdr*-1 coding gene leading to amino acid changes in *Pfmdr1* have different consequences on parasite’s sensitivity to anti-malarial drugs. Several *Pfmdr1* single nucleotide polymorphisms have been reported whereby N86Y, Y184F, S1034C, N1042D and D1246Y are the most common. *Pfmdr1* 86Y mutation is associated with chloroquine (CQ) and amodiaquine (AQ) resistance [2-4], while 1034C, 1042D and 1246Y mutations have been reported to confer resistance against quinine (QN) and increased susceptibility to mefloquine (MQ), halofantrine (HF) and artemisinin [5-7]. Furthermore, the 86Y and 1246Y are highly associated with decreased sensitivity to artesunate-amodiaquine (AS-AQ), while the wild types N86 and D1246 are linked to artemether-lumefantrine (ALu) resistance [8-10]. Recent studies have shown that the combination of N86, 184 F, and D1246 forming a haplotype “NFD” lead to decreased susceptibility to ALu and that treatment with ALu selects for such haplotype [11,12]. Furthermore, an increase of asexual parasites and gametocytes harboring *Pfmdr1* NFD haplotype in patients treated with ALu was linked with treatment failure [12].

In Tanzania, ALu was adopted as first-line treatment drug in December 2006 [13]. A recent study in Korogwe, Tanga region reported increase of N86 from 25% to 59% and 184 F from 10% to 30% in 2006 to 2010 [14]. Another study in Igongwe, Mwanza pointed out an increase of N86 in samples collected post ALu treatment as compared with pretreatment samples; from 6.3 to 42,1% [15]. Also in Bagamoyo Pwani region, Malmberg and colleagues reported increase from 10 to 37% of the NFD haplotype from 2006 to 2011 [16]. A similar selection of NFD by ALu was observed in Mozambique [17]. In Kenya the Y184F was associated with high artemisinin IC_50_ levels in ex-vivo drug sensitivity assays while the wild type N86 was associated with high MQ IC_50_[10]. Furthermore, *P. falciparum* parasites carrying NFD haplotype were able to withstand 15-fold higher blood lumefantrine levels than those with YYY (86Y-Y184-1246Y) haplotype [18]. Recently, ACT resistance associated K13 propeller protein mutations selected through increasing drug pressure in laboratory strains and subsequently found in field isolates from South-East Asia were reported [19]. Together with the *Pfmdr1* ALu-associated haplotypes the K13 polymorphism is evidence of emerging tolerance to ACT and calls for continuous monitoring surveillance studies. Following five years of ALu treatment policy implementation in Tanzania there is scarcity of information on current status of ACT markers of resistance. Of the studies reported to-date most were conducted a few years around the official adoption of ALu in the country (from 2003–2009), while the few most recent had inadequate sample size or did not cover the NFD haplotype with exception of one study [16] conducted in 2010. This study reports on the current status of the *Pfmdr1* NFD haplotype in six regions of Tanzania which can be used as a baseline status for future studies in predicting the trends and for monitoring ALu efficacy.

## Methods

### Description of study subjects and study sites

Samples used in this study were obtained through collaboration with ongoing studies in six regions of mainland Tanzania between June 2010 and August 2011. Except for the Coastal region where the samples involved pregnant women attending the Kibiti health centre for antenatal care, all other samples were collected from all-age groups. Finger prick blood on filter paper (Whatman-3) or malaria rapid diagnostic test (RDT) (Paracheck, Orchid Biomedical Systems, India) (Mwanza samples only) from febrile patients attending to various health facilities in the respective regions were collected after patient’s or children’s guardians had consented for use of their blood samples for malarial genetic studies. The study sites (with their respective number of samples in brackets) include Mwanza (Misungwi district, n = 107) and Kagera (Muleba district, n = 129) around Lake Victoria in the north-western zone, Tanga (Bondo village, n = 94) in north-eastern zone, Mtwara (Tandahimba and Mtwara-Urban, n = 70) and Coastal Region (Kibiti-Rufiji, n = 144) in south-eastern zone and Mbeya (Kyela and Rungwe districts, n = 143) in the south-western zone.

### DNA extraction and genotyping of the *Pfmdr1* gene

Malaria-positive RTDs or dried filter paper blood spots from microscopically confirmed cases were stored in desiccants at room temperature. Malaria parasite DNA was extracted using chelex-100 method as described previously [20]. Genotyping for *Pfmdr1* was performed using PCR-RFLP-methods described elsewhere [7,21]. In brief, PCR products were digested with *Apo*I and *Afl-*III which recognize the 86 N and (86Y) respectively, *Dra-*I which recognises the 184Y and *Eco*RV which recognises 1246Y. Endonuclease digest products were eluted on 2.5% agarose gel (Amasham Biosciences, Sweden) stained with ethidium bromide (Sigma Aldrich, USA) and visualized under ultraviolet light. PCR reagents and restriction endonucleases were purchased from New England Biolabs (NEB inc., Ipswich, MA, USA). Primers were purchased from Biolegio (Biolegio Inc., The Netherlands). Prevalence was calculated by adding the number of samples carrying mixed infections to both wild-type allele and mutant allele, thereby obtaining a new ‘n’ (which includes the mixed infections twice). Prevalence of wild-type and mutant allele was then calculated as the percentage of wild-type plus mixed infection or mutants plus mixed infection out of the new ‘n’. For the haplotype analysis the mixed infections were however excluded.

Statistical analysis was performed using Pearson Chi-squire (SPSS version 16) and Fisher’s exact (FE) test. The study received ethical approval from the Kilimanjaro Christian Medical University College ethical board subsequent to the National IRB (NIMR) approval obtained in the collaborating projects.

## Results

**Figure 1:**
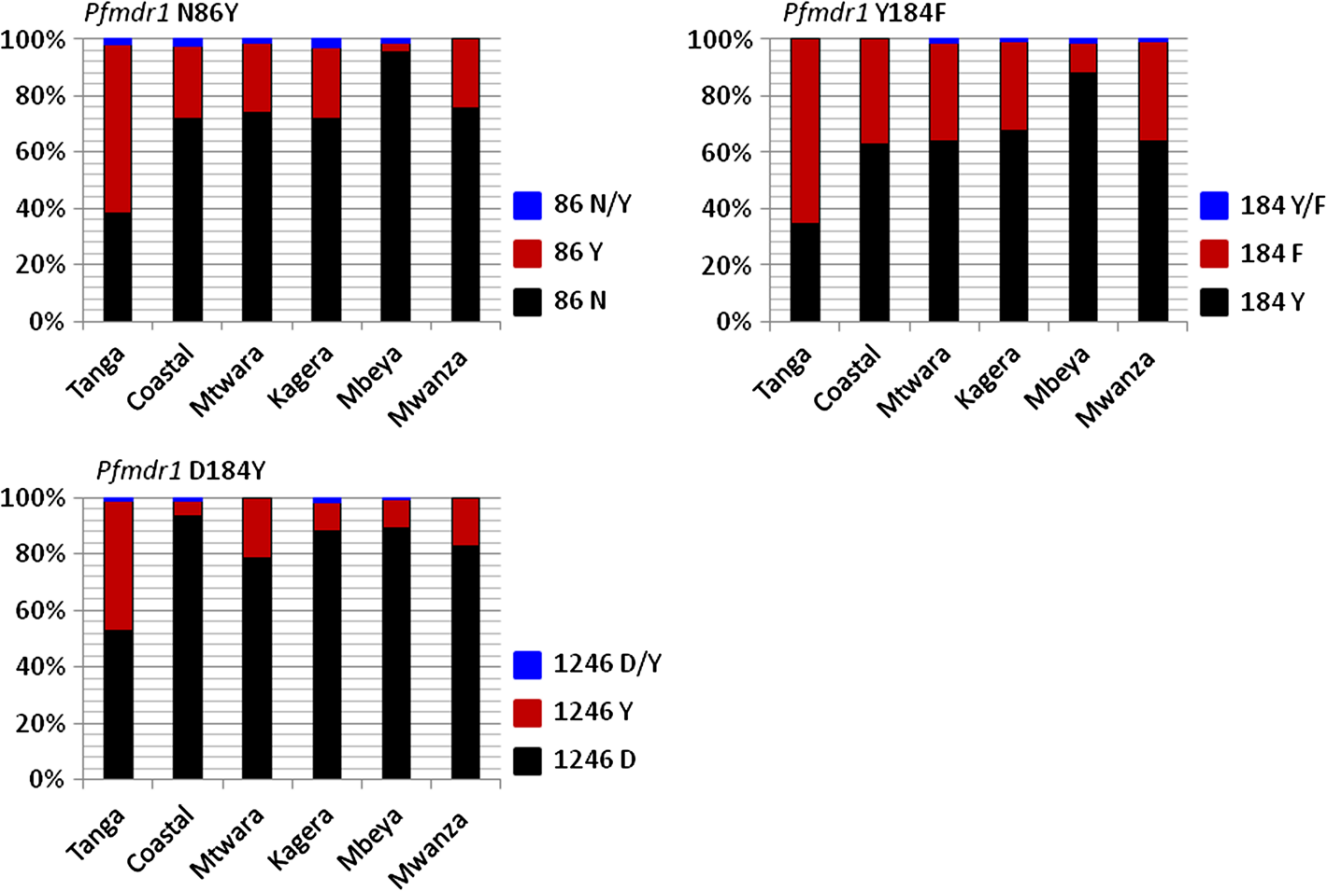
**Prevalence of the *Pfmdr1 *N86Y, Y184F, and D1246Y polymorphisms by region in Tanzania.** Shown in black, Wild-types; brick-red: Mutants and Blue: mixed genotypes.

Out of the 687 samples, 644 (93.7), 641 (93.3) and 646 (94%) were successfully genotyped for *Pfmdr1* N86Y, Y184F and D1246Y SNPs respectively. There was statistically significant difference in the distribution of individual *Pfmdr1* polymorphisms among the regions; N86Y (*χ*^2^ = 91.0, p <0.0001), Y184F (*χ*^2^ = 68.4, p < 0.0001) and D1246Y (*χ*^2^ = 73.7, p < 0.0001). Tanga region had the highest prevalence of mutant alleles in all codons while Mbeya had the highest prevalence of wild type alleles for N86Y and Y184F (Table 1 and Figure 1).

**Table 1:** **Distribution of *Pfmdr1 *** **single nucleotide polymorphisms in Tanzania**

**Regions**	***Pfmdr1 *** **polymorphisms**											
	**N86Y**				**Y184F**				**D1246Y**			
	**N**	**Y**	**N/Y**	**Y**	**Y**	**F**	**Y/F**	**F**	**D**	**Y**	**D/Y**	**Y**
	**n (%)**	**n (%)**	**n (%)**	**Prevalence (%)**	**n (%)**	**n (%)**	**n (%)**	**Prevalence (%)**	**n (%)**	**n (%)**	**n (%)**	**Prevalence (%)**
Tanga	36 (38.3)	56 (59.6)	2 (2.1)	58 (60.4)	32 (34.8)	60 (65.2)	0 (0)	60 (65.2)	45 (52.9)	39 (45.9)	1 (1.2)	40 (46.5)
Coastal	93 (72.1)	33 (25.6)	3 (2.3)	36 (27.3)	85 (63.0)	50 (37.0)	0 (0)	50 (37.0)	134 (93.7)	7 (4.9)	2 (1.4)	9 (6.2)
Mtwara	49 (74.2)	16 (24.2)	1 (1.5)	17 (25.4)	43 (64.2)	23 (34.3)	1 (1.5)	24 (35.3)	55 (78.6)	15 (21.4)	0 (0)	15 (21.4)
Kagera	90 (72.0)	31 (24.8)	4 (3.2)	35 (27.1)	82 (67.8)	38 (31.4)	1 (0.8)	39 (32.6)	112 (88.2)	13 (10.2)	2 (1.6)	15 (11.6)
Mbeya	129 (95.6)	4 (3.0)	2 (1.5)	6 (4.4)	111 (88.0)	13 (10.3)	2 (1.6)	15 (11.7)	119 (89.5)	13 (9.8)	1 (0.8)	14 (10.4)
Mwanza	72 (77.1)	23 (22.9)	0 (0)	23 (24.2)	64 (64.0)	35 (35.0)	1 (1.0)	36 (35.6)	73 (83.0)	15 (17.0)	0 (0)	15 (23.6)
Total	469 (72.8)	163 (25.3)	12 (1.8)		417 (65)	219 (34.2)	5 (0.7)		538 (83)	102 (15.7)	6 (0.9)	

### Haplotype analysis

**Figure 2:**
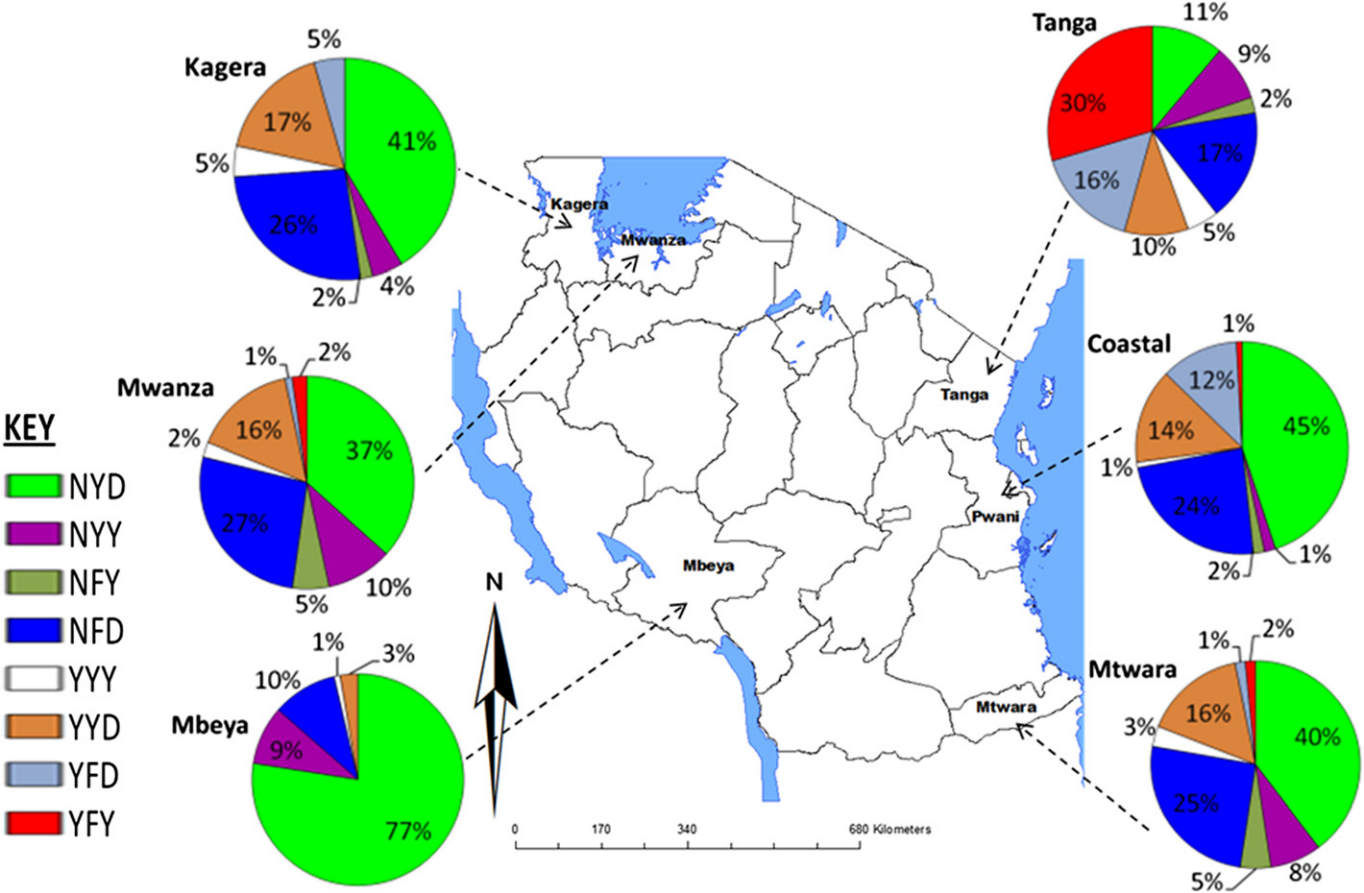
**Prevalence of *Pfmdr1 *N86Y, Y184F, and D1246Y haplotypes in Tanzania.** The number of samples analyzed per region were Tanga (n = 81), Coastal (n = 118), Mtwara (n = 70), Mbeya (n = 111), Mwanza (n = 90) and Kagera (n = 111).

When the SNPs were constructed into codon 86-184-1246 haplotypes, eight haplotypes were detected among 578 of the samples, omitting samples that had mixed genotype infections (Table 2) and those that could not be genotyped for all the three SNPs. Of these haplotypes, the most common were NYD (43.6%), NFD (21.1%) and YYD (12.3%) haplotypes (Figure 2). A minor haplotype YFY (4.8%), was almost exclusively present in Tanga region (85.7% of total YFY haplotypes) compared to other regions and was the most prevalent (29.6%) of the eight haplotypes in that region. Conversely, the NYD was the most prevalent in all other regions, with a markedly high prevalence in Mbeya (77.5%) compared to the other regions (Figure 2). When comparing individual haplotypes against the regions, each haplotype varied significantly between the regions (p < 0.05). However, when Mbeya with exceptionally high wild-type haplotype (77.5%) was excluded, the NFD distribution did not vary between the regions (*χ*^2^ = 2.3, p = 0.512). Furthermore, when both Mbeya and Tanga were excluded from the analysis, all the common haplotypes did not vary significantly among the regions (YYD: *χ*^2^ = 0.32, p = 0.952; NYD: *χ*^2^ = 1.498, p = 0.683; NFD: *χ*^2^ = 0.28, p = 0.964 and YFY: FE = 2.77, p = 0.462). Mbeya and Tanga regions were, therefore, exceptional with Tanga having the most mutant alleles at the three codons while Mbeya had the most wildtypes in two of the three.

**Table 2:** **Prevalence of the *Pfmdr1 *** **haplotypes in six regions of Tanzania**

**Regions**	***Pfmdr1 *** **haplotypes**								**Total (N)**
	**NYD**	**NYY**	**NFY**	**NFD**	**YYY**	**YYD**	**YFD**	**YFY**	
	**n (%)**	**n (%)**	**n (%)**	**n (%)**	**n (%)**	**n (%)**	**n (%)**	**n (%)**	
**Tanga**	9 (11.1)	7 (8.6)	2 (2.5)	14 (17.3)	4 (4.9)	8 (9.9)	13 (16.0)	24 (29.6)	**81**
**Coastal**	53 (44.9)	2 (1.7)	2 (1.7)	28 (23.7)	1 (0.8)	17 (14.4)	14 (11.9)	1 (0.8)	**118**
**Mtwara**	25 (39.7)	5 (7.9)	3 (4.8)	16 (25.4)	2 (3.2)	10 (15.9)	1 (1.6)	1 (1.6)	**63**
**Mbeya**	86 (77.5)	10 (9.0)	0 (0.0)	11 (9.9)	1 (0.9)	3 (2.7)	0 (0.0)	0 (0.0)	**111**
**Kagera**	46 (41.4)	5 (4.5)	2 (1.8)	29 (26.1)	5 (4.5)	19 (17.1)	5 (4.5)	0 (0.0)	**111**
**Mwanza**	33 (36.7)	9 (10.0)	5 (5.6)	24 (26.7)	2 (2.2)	14 (15.6)	1 (1.1)	2 (2.2)	**90**
**Total N (%)**	**252 (43.6)**	**38 (6.6)**	**14 (2.4)**	**122 (21.1)**	**15 (2.6)**	**71 (12.3)**	**34 (5.9)**	**28 (4.8)**	**578**

## Discussion

Molecular markers are useful predictors of emerging or existing levels of resistance to anti-malarial drugs. The surveillance of these markers have proven important during recent years where reports on the molecular marker for chloroquine (CQ) resistance; *Pfcrt* have shown recovery of CQ sensitivity in Mozambique and Tanzania [22-24]. Furthermore, accumulation of mutations in the genes *Pfdhfr* and *Pfdhps* associated to sulphadoxine-pyrimethamine (SP) resistance have recently been shown to culminate with the emergence of sextuple *Pfdhfr* and *Pfdhps* mutants [25,26]. These super-resistant mutants render intermittent preventive treatment of pregnant women (IPTp) using SP redundant in places such as in Tanga where high prevalence of such mutants have been documented [27]. In this study, variation in the distribution of *Pfmdr1* polymorphisms among regions in Tanzania is reported. The overall prevalence of single SNPs and as well, the resulting triple 86-184-1246 haplotype YFY haplotype was highest in Tanga. Interestingly, this coincides with highest prevalence of SP resistance markers also documented in Tanga region [25,28,29]. The haplotype YFY is linked to AQ and CQ resistance [21]. On the other hand high prevalence of NYD haplotype was highest in Mbeya region. This made Tanga and Mbeya regions different from the rest of the studied regions. While there is no clear explanation for Mbeya, a general very high malaria transmission thus high use of anti-malarials especially in early 1980s and 1990s may have led to a particularly high selection pressure for resistant parasites in Tanga relative to other places in Tanzania [30,31]. In a recent survey on availability of anti-malarials in Muheza Tanga, AQ and SP were still available in private shops and used by the local population for malaria self-medication [32]. Continued use of AQ in the study area or neighbourhood may account for the observed high YFY haplotype. Also these findings point to a possible low adherence to the ALu treatment policy in Tanga relative to other regions.

The NFD did not vary between five of the regions. These results show homogeneity in *Pfmdr1* haplotypes distribution, which suggests similar selection pressure throughout the country, indicative of homogeneity in ALu policy implementation in Tanzania. ALu has been shown to select for the NFD haplotype, where the prevalence of 86Y and 1246Y mutations has been decreasing while the 184 F has been increasing [11,14,17]. In this study, low prevalence of mutations 86Y and 1246Y were observed relative to 184 F. Similar findings elsewhere in East and West Africa have been reported where ALu is the treatment policy [33-35]. In recent in-vitro studies done using parasite isolates in Senegal and South East Asia, the 86Y and 1246Y were associated with high CQ, AQ and MQ inhibitory concentrations (IC_50_) whereas the 184 F was associated with high artemisinin IC_50_ values [36,37]. Furthermore, in Cambodian samples the prevalence of the 184 F mutation selectively increased after ACT pressure [38]. These reports are suggestive of some overlap in mechanism of ACT resistance between South-East Asia and Africa and that these molecular markers can serve as universal tools for ACT resistance monitoring.

## Conclusions

This is the first country-wide survey on *Pfmdr1* mutations associated with ACT resistance. Distribution of *Pfmdr1* mutations at codons 86, 184 and 1246 varies throughout Tanzanian regions. There is homogeneity in distribution of common *Pfmdr1* haplotypes in four out of six regions of Tanzania which may reflects homogeneity in countrywide implementation of ALu policy. The overall prevalence of NFD haplotype claimed to be associated with emerging ALu tolerance ranges from 17 to 26% among other haplotypes. With continuation of ALu as first-line drug and in the absence of CQ and AQ, this haplotype is expected to keep rising. There is need for continued pharmacovigilance studies in order to predict early parasite tolerance to the drug.

## Competing interests

The authors declare that they have no competing interests.

## Authors’ contributions

RAK conceived the idea, designed the study, analysed the data and wrote the manuscript. PP participated in study design, performed the experiments, participated in interpreting the data and drafted the manuscript. RDK participated in performing the experiments and in manuscript writing. AK supervised sample collection in the field and revised the manuscript. MvS and JC participated in analysing the data and revised the manuscript. CR and MA participated in overall interpretation of the results and in writing the manuscript. All authors read and approved the final version of the manuscript.

## References

[B1] Cowman AF Karcz S Galatis D Culvenor JG A P-glycoprotein homologue of *Plasmodium falciparum* is localized on the digestive vacuole J Cell Biol 1991 113 1033 1042 1674943 10.1083/jcb.113.5.1033 PMC2289011

[B2] Duraisingh MT Drakeley CJ Muller O Bailey R Snounou G Targett GA Greenwood BM Warhurst DC Evidence for selection for the tyrosine-86 allele of the pfmdr 1 gene of *Plasmodium falciparum* by chloroquine and amodiaquine Parasitology 1997 114 205 211 9075340 10.1017/s0031182096008487

[B3] Folarin OA Bustamante C Gbotosho GO Sowunmi A Zalis MG Oduola AM Happi CT In vitro amodiaquine resistance and its association with mutations in pfcrt and pfmdr1 genes of *Plasmodium falciparum* isolates from Nigeria Acta Trop 2011 120 224 230 21920347 10.1016/j.actatropica.2011.08.013 PMC3214618

[B4] Tinto H Guekoun L Zongo I Guiguemde RT D’Alessandro U Ouedraogo JB Chloroquine-resistance molecular markers (Pfcrt T76 and Pfmdr-1 Y86) and amodiaquine resistance in Burkina Faso Trop Med Int Health 2008 13 238 240 18304270 10.1111/j.1365-3156.2007.01995.x

[B5] Lekostaj JK Amoah LE Roepe PD A single S1034C mutation confers altered drug sensitivity to PfMDR1 ATPase activity that is characteristic of the 7G8 isoform Mol Biochem Parasitol 2008 157 107 111 18006157 10.1016/j.molbiopara.2007.09.008 PMC2211713

[B6] Sidhu AB Valderramos SG Fidock DA Pfmdr1 mutations contribute to quinine resistance and enhance mefloquine and artemisinin sensitivity in *Plasmodium falciparum* Mol Microbiol 2005 57 913 926 16091034 10.1111/j.1365-2958.2005.04729.x

[B7] Humphreys GS Merinopoulos I Ahmed J Whitty CJ Mutabingwa TK Sutherland CJ Hallett RL Amodiaquine and artemether-lumefantrine select distinct alleles of the *Plasmodium falciparum* mdr1 gene in Tanzanian children treated for uncomplicated malaria Antimicrob Agents Chemother 2007 51 991 997 17194834 10.1128/AAC.00875-06 PMC1803116

[B8] Pickard AL Wongsrichanalai C Purfield A Kamwendo D Emery K Zalewski C Kawamoto F Miller RS Meshnick SR Resistance to antimalarials in Southeast Asia and genetic polymorphisms in pfmdr1 Antimicrob Agents Chemother 2003 47 2418 2423 12878499 10.1128/AAC.47.8.2418-2423.2003 PMC166057

[B9] Reed MB Saliba KJ Caruana SR Kirk K Cowman AF Pgh1 modulates sensitivity and resistance to multiple antimalarials in *Plasmodium falciparum* Nature 2000 403 906 909 10706290 10.1038/35002615

[B10] Mwai L Kiara SM Abdirahman A Pole L Rippert A Diriye A Bull P Marsh K Borrmann S Nzila A In vitro activities of piperaquine, lumefantrine, and dihydroartemisinin in Kenyan *Plasmodium falciparum* isolates and polymorphisms in pfcrt and pfmdr1 Antimicrob Agents Chemother 2009 53 5069 5073 19770282 10.1128/AAC.00638-09 PMC2786317

[B11] Baliraine FN Rosenthal PJ Prolonged selection of pfmdr1 polymorphisms after treatment of falciparum malaria with artemether-lumefantrine in Uganda J Infect Dis 2011 204 1120 1124 21881128 10.1093/infdis/jir486 PMC3164433

[B12] Happi CT Gbotosho GO Folarin OA Sowunmi A Hudson T O’Neil M Milhous W Wirth DF Oduola AM Selection of *Plasmodium falciparum* multidrug resistance gene 1 alleles in asexual stages and gametocytes by artemether-lumefantrine in Nigerian children with uncomplicated falciparum malaria Antimicrob Agents Chemother 2009 53 888 895 19075074 10.1128/AAC.00968-08 PMC2650543

[B13] Njau JD Goodman CA Kachur SP Mulligan J Munkondya JS McHomvu N Abdulla S Bloland P Mills A The costs of introducing artemisinin-based combination therapy: evidence from district-wide implementation in rural Tanzania Malar J 2008 7 4 18179716 10.1186/1475-2875-7-4 PMC2249587

[B14] Thomsen TT Ishengoma DS Mmbando BP Lusingu JP Vestergaard LS Theander TG Lemnge MM Bygbjerg IC Alifrangis M Prevalence of single nucleotide polymorphisms in the *Plasmodium falciparum* multidrug resistance gene (Pfmdr-1) in Korogwe District in Tanzania before and after introduction of artemisinin-based combination therapy Am J Trop Med Hyg 2011 85 979 983 22144430 10.4269/ajtmh.2011.11-0071 PMC3225174

[B15] Kamugisha E Jing S Minde M Kataraihya J Kongola G Kironde F Swedberg G Efficacy of artemether-lumefantrine in treatment of malaria among under-fives and prevalence of drug resistance markers in Igombe-Mwanza, north-western Tanzania Malar J 2012 11 58 22369089 10.1186/1475-2875-11-58 PMC3305412

[B16] Malmberg M Ngasala B Ferreira PE Larsson E Jovel I Hjalmarsson A Petzold M Premji Z Gil JP Bjorkman A Martensson A Temporal trends of molecular markers associated with artemether-lumefantrine tolerance/resistance in Bagamoyo district, Tanzania Malar J 2013 12 103 23506218 10.1186/1475-2875-12-103 PMC3732084

[B17] Thomsen TT Madsen LB Hansson HH Tomas EV Charlwood D Bygbjerg IC Alifrangis M Rapid selection of *Plasmodium falciparum* chloroquine resistance transporter gene and multidrug resistance gene-1 haplotypes associated with past chloroquine and present artemether-lumefantrine use in Inhambane District, southern Mozambique Am J Trop Med Hyg 2013 88 536 541 23382159 10.4269/ajtmh.12-0525 PMC3592537

[B18] Malmberg M Ferreira PE Tarning J Ursing J Ngasala B Bjorkman A Martensson A Gil JP *Plasmodium falciparum* drug resistance phenotype as assessed by patient antimalarial drug levels and its association with pfmdr1 polymorphisms J Infect Dis 2013 207 842 847 23225895 10.1093/infdis/jis747 PMC3563306

[B19] Ariey F Witkowski B Amaratunga C Beghain J Langlois AC Khim N Kim S Duru V Bouchier C Ma L Lim P Leang R Duong S Sreng S Suon S Chuor CM Bout DM Menard S Rogers WO Genton B Fandeur T Miotto O Ringwald P Le BJ Berry A Barale JC Fairhurst RM Benoit-Vical F Mercereau-Puijalon O A molecular marker of artemisinin-resistant *Plasmodium falciparum* malaria Nature 2014 505 50 55 24352242 10.1038/nature12876 PMC5007947

[B20] Polski JM Kimzey S Percival RW Grosso LE Rapid and effective processing of blood specimens for diagnostic PCR using filter paper and Chelex-100 Mol Pathol 1998 51 215 217 9893748 10.1136/mp.51.4.215 PMC395639

[B21] Duraisingh MT Roper C Walliker D Warhurst DC Increased sensitivity to the antimalarials mefloquine and artemisinin is conferred by mutations in the pfmdr1 gene of *Plasmodium falciparum* Mol Microbiol 2000 36 955 961 10844681 10.1046/j.1365-2958.2000.01914.x

[B22] Kublin JG Cortese JF Njunju EM Mukadam RA Wirima JJ Kazembe PN Djimde AA Kouriba B Taylor TE Plowe CV Reemergence of chloroquine-sensitive *Plasmodium falciparum* malaria after cessation of chloroquine use in Malawi J Infect Dis 2003 187 1870 1875 12792863 10.1086/375419

[B23] Laufer MK Thesing PC Eddington ND Masonga R Dzinjalamala FK Takala SL Taylor TE Plowe CV Return of chloroquine antimalarial efficacy in Malawi N Engl J Med 2006 355 1959 1966 17093247 10.1056/NEJMoa062032

[B24] Mohammed A Ndaro A Kalinga A Manjurano A Mosha JF Mosha DF van-Zwetselaar M Koenderink JB Mosha FW Alifrangis M Reyburn H Roper C Kavishe RA Trends in chloroquine resistance marker, Pfcrt-K76T mutation ten years after chloroquine withdrawal in Tanzania Malar J 2013 12 415 24225406 10.1186/1475-2875-12-415 PMC3830541

[B25] Gesase S Gosling RD Hashim R Ord R Naidoo I Madebe R Mosha JF Joho A Mandia V Mrema H Mapunda E Savael Z Lemnge M Mosha FW Greenwood B Roper C Chandramohan D High resistance of *Plasmodium falciparum* to sulphadoxine/pyrimethamine in northern Tanzania and the emergence of dhps resistance mutation at Codon 581 PLoS One 2009 4 e4569 19238219 10.1371/journal.pone.0004569 PMC2644264

[B26] Naidoo I Roper C Mapping ‘partially resistant’, ‘fully resistant’, and ‘super resistant’ malaria Trends Parasitol 2013 29 505 515 24028889 10.1016/j.pt.2013.08.002

[B27] Minja DT Schmiegelow C Mmbando B Bostrom S Oesterholt M Magistrado P Pehrson C John D Salanti A Luty AJ Lemnge M Theander T Lusingu J Alifrangis M *Plasmodium falciparum* mutant haplotype infection during pregnancy associated with reduced birthweight, Tanzania Emerg Infect Dis 2013 19 9 10.3201/eid1909.130133 PMC3810920 23969132

[B28] Matondo SI Temba GS Kavishe AA Kauki JS Kalinga A van-Zwetselaar M Reyburn H Kavishe RA High levels of sulphadoxine-pyrimethamine resistance Pfdhfr-Pfdhps quintuple mutations: a cross sectional survey of six regions in Tanzania Malar J 2014 13 152 24751352 10.1186/1475-2875-13-152 PMC3998221

[B29] Alifrangis M Lusingu JP Mmbando B Dalgaard MB Vestergaard LS Ishengoma D Khalil IF Theander TG Lemnge MM Bygbjerg IC Five-year surveillance of molecular markers of *Plasmodium falciparum* antimalarial drug resistance in Korogwe District, Tanzania: accumulation of the 581G mutation in the *P. falciparum* dihydropteroate synthase gene Am J Trop Med Hyg 2009 80 523 527 19346369

[B30] Yavo W Faye B Kuete T Djohan V Oga SA Kassi RR Diatta M Ama MV Tine R Ndiaye JL Evi JB Same-Ekobo A Faye O Kone M Multicentric assessment of the efficacy and tolerability of dihydroartemisinin-piperaquine compared to artemether-lumefantrine in the treatment of uncomplicated *Plasmodium falciparum* malaria in sub-Saharan Africa Malar J 2011 10 198 21774826 10.1186/1475-2875-10-198 PMC3164625

[B31] Schneider AG Premji Z Felger I Smith T Abdulla S Beck HP Mshinda H A point mutation in codon 76 of pfcrt of *P. falciparum* is positively selected for by Chloroquine treatment in Tanzania Infect Genet Evol 2002 1 183 189 12798014 10.1016/s1567-1348(01)00021-1

[B32] Ringsted FM Massawe IS Lemnge MM Bygbjerg IC Saleability of anti-malarials in private drug shops in Muheza, Tanzania: a baseline study in an era of assumed artemisinin combination therapy (ACT) Malar J 2011 10 238 21843328 10.1186/1475-2875-10-238 PMC3167767

[B33] Conrad MD Leclair N Arinaitwe E Wanzira H Kakuru A Bigira V Muhindo M Kamya MR Tappero JW Greenhouse B Dorsey G Rosenthal PJ Comparative impacts over 5 years of artemisinin-based combination therapies on *P. falciparum* polymorphisms that modulate drug sensitivity in Ugandan children J Infect Dis 2014 210 344 353 24610872 10.1093/infdis/jiu141 PMC4110461

[B34] Duah NO Matrevi SA de Souza DK Binnah DD Tamakloe MM Opoku VS Onwona CO Narh CA Quashie NB Abuaku B Duplessis C Kronmann KC Koram KA Increased pfmdr1 gene copy number and the decline in pfcrt and pfmdr1 resistance alleles in Ghanaian *Plasmodium falciparum* isolates after the change of anti-malarial drug treatment policy Malar J 2013 12 377 24172030 10.1186/1475-2875-12-377 PMC3819684

[B35] Gadalla NB Abdallah TM Atwal S Sutherland CJ Adam I Selection of pfdhfr/pfdhps alleles and declining artesunate/sulphadoxine-pyrimethamine efficacy against *Plasmodium falciparum* eight years after deployment in eastern Sudan Malar J 2013 12 255 23870667 10.1186/1475-2875-12-255 PMC3720549

[B36] Na-Bangchang K Muhamad P Ruaengweerayut R Chaijaroenkul W Karbwang J Identification of resistance of *Plasmodium falciparum* to artesunate-mefloquine combination in an area along the Thai-Myanmar border: integration of clinico-parasitological response, systemic drug exposure, and in vitro parasite sensitivity Malar J 2013 12 263 23898808 10.1186/1475-2875-12-263 PMC3737112

[B37] Van TD Dieye B Valim C Daniels RF Sene PD Lukens AK Ndiaye M Bei AK Ndiaye YD Hamilton EJ Ndir O Mboup S Volkman SK Wirth DF Ndiaye D Changes in drug sensitivity and anti-malarial drug resistance mutations over time among *Plasmodium falciparum* parasites in Senegal Malar J 2013 12 441 24314037 10.1186/1475-2875-12-441 PMC3924193

[B38] Vinayak S Alam MT Sem R Shah NK Susanti AI Lim P Muth S Maguire JD Rogers WO Fandeur T Barnwell JW Escalante AA Wongsrichanalai C Ariey F Meshnick SR Udhayakumar V Multiple genetic backgrounds of the amplified *Plasmodium falciparum* multidrug resistance (pfmdr1) gene and selective sweep of 184 F mutation in Cambodia J Infect Dis 2010 201 1551 1560 20367478 10.1086/651949 PMC3096139

